# Influence of Different Deep Eutectic Solvents and Plant Extracts on Antioxidant, Mechanical, and Color Properties of Alginate Film

**DOI:** 10.3390/polym16142084

**Published:** 2024-07-22

**Authors:** Jolanta Kowalonek, Malo Hamieau, Aleksandra Szydłowska-Czerniak

**Affiliations:** 1 Department of Biomedical and Polymer Chemistry, Faculty of Chemistry, Nicolaus Copernicus University in Toruń, Gagarina 7, 87-100 Toruń, Poland; 2 Univ Rennes, IUT de Rennes, F-35000 Rennes, France; malo.hamieau@etudiant.univ-rennes1.fr; 3 Department of Analytical Chemistry and Applied Spectroscopy, Faculty of Chemistry, Nicolaus Copernicus University in Toruń, Gagarina 7, 87-100 Toruń, Poland; olasz@umk.pl

**Keywords:** alginate film, chokeberry, lemon balm, deep eutectic solvents, antioxidant capacity, mechanical properties, color

## Abstract

Eco-friendly functional alginate films with plant extracts (chokeberry pomace (ChP) or lemon balm (LB) herb) were obtained. Moreover, deep eutectic solvents (DESs) based on choline chloride, glucose, and betaine were used to acquire the active substances from plant materials. The films were tested regarding the antioxidant, mechanical, and color properties. The results revealed that the films’ antioxidant capacities (AC) depended on the extract type and DES used, namely AC values for alginate films with LB were higher than those with ChP. Moreover, the results of the films’ mechanical properties depended only on the DES, which acted as a plasticizer in most cases. Furthermore, the color analysis of the studied films showed a dependence on the type of extract and DES. The lightness (L*) was influenced only by the DES type, while the solvent and extract type affected the a* and b* values. Our results show that the films can be applied as active packaging for food products.

## 1. Introduction

The problem associated with using petroleum-based plastic products is the creation of large amounts of polymer waste that do not decompose and accumulate in landfills, which is not indifferent to the natural environment. One-third of synthetic polymer applications are packaging [1]. Lately, seeking substitutes for fossil-derived polymers has been the subject of studies. Packaging based on natural polymers is an excellent alternative to synthetic polymers in food packaging, especially for perishable food such as fruits, vegetables, cheese, etc. [2,3,4]. Biopolymers, such as polysaccharides, proteins, and lipids, are naturally abundant, non-toxic, and biodegradable. Among polysaccharides, alginates are the substitutes for non-degradable synthetic polymers. However, alginates and all polysaccharides form brittle and stiff films due to the strong interaction between biomacromolecules. Therefore, a plasticizer is added to make the film flexible and elastic owing to the reduction in the intermolecular forces between biopolymer chains and increasing free volume [1,5,6]. Polyols, especially glycerol, have been applied as plasticizers. Deep eutectic solvents (DESs) have recently become popular instead of glycerol, especially in the food and pharmaceutical industries [7,8].

DESs are mixtures of two or three components, each acting as either a hydrogen bond donor (HBD) or a hydrogen bond acceptor (HBA). Importantly, DESs are easy to prepare and cost-effective; many are biodegradable and unreactive with water. Recently, DESs have been widely used in the food sector mainly (1) as “green” solvents to extract bioactive compounds, macromolecules (proteins, carbohydrates, lipids), undesired compounds (heavy metals, minerals), hazardous chemicals, etc., (2) as plasticizers to improve the mechanical properties (flexibility, elasticity) of food packaging films, (3) to encapsulate bioactive compounds with low bioavailability, (4) as anti-freezing agents for the frozen food industries, and (5) as enhancers of the organoleptic properties of food products [9,10,11].

Interestingly, in the food packaging area, DESs can act simultaneously as the pretreatment solvents of biopolymers, plasticizers, antioxidant and/or antimicrobial agents, and solvents to extract the bioactive compounds added to film matrices.

In recent years, different DESs have been used to modify the mechanical properties of packaging films based on chitosan [12,13], polysaccharides [7], methylcellulose [14], gelatine [15], and pectin [16].

Beyond the excellent plasticizing effect of various DESs on packaging films, these “green” solvents can enhance the antioxidant capacities (AC) of films due to (1) the unique antioxidant properties of their components, HBA and HBD, and (2) the ability to extract the hydrophobic and hydrophilic antioxidant compounds from natural sources, which can add to active packaging. The presence of red grape skin and DES (choline chloride (ChCl):glucose (Glu), 2:1) in polyvinyl alcohol/cellulose nanocrystal film caused an increase in its antioxidant potential by 240.1%, measured via a 2,2–diphenyl–1–picrylhydrazyl (DPPH) assay [17]. Moreover, astaxanthin recovered from shrimp wastes using ultrasound-assisted extraction (UAE) and natural ChCl-based DESs enhanced the AC of chitosan films determined via DPPH and 2,2′–azino–bis(3-ethylbenzothiazoline–6–sulfonic acid) (ABTS) methods [18]. Unexpectedly, the extracts obtained from *L. chequen* fruit and lavender flowers prepared via UAE using five different glycerol (Gly)-based DESs as well as a mixture of ChCl:Gly (1:2) and introduced to carrageenan and pectin films, respectively, insignificantly affected their antioxidant features measured by the bleaching of the DPPH and ABTS radicals [16,19].

These selective and probably complex matrix interferences are critical and require future explanations for broadening the use of DESs in developing new active and biodegradable food packaging. 

Sodium alginate is a biopolymer suitable for packaging. It is a linear anionic polysaccharide composed of β-D-mannuronate and α–L–guluronate units linked by β–(1–4) glycosidic bonds. The contribution and distribution of these units in macromolecules determine the properties of this biopolymer, such as viscosity, gel formation, and water uptake. Alginate chains rich in mannuronate units are flexible and do not form a gel, while the chains containing a predominance of guluronate units have a zigzag structure, are stiff, and can create a gel with divalent ions. Generally, alginate chains are stiff macromolecules because they are built of six-membered sugar rings [1,20].

Seaweeds are the source of alginates, which are available on the market as white or yellow powder. Sodium alginate has found many various applications. One of the applications of this polysaccharide is food packaging due to its excellent film-forming properties; however, antioxidant and antibacterial compounds are needed to make such films active. There are many publications describing the successful usage of alginate as packaging film, for instance, an alginate-based film with cinnamaldehyde as active compounds for strawberry preservation [21], alginate with essential oils from *A. herba alba* Asso, *O. basilicum* L., *M. pulegium* L., and *R. officinalis* for cheese preservation [22], alginate with silver nanoparticles and lemon grass essential oil for cheese protection [3], and an alginate edible coating with an olive leaf extract to prolong the shelf life of sweet cherries [23].

On the other hand, bioactive compounds such as simple and functionalized phenolics, flavonoids, tannins, organic acids, anthraquinone, and vitamins derived from plant extracts and essential oils have potent antioxidant, metal chelating, reducing power, and antimicrobial properties [24,25]. Therefore, the enrichment of polymeric materials into plant extracts rich in bioactive natural components introduces additional functionalities such as antioxidant activity, antimicrobial efficacy by inhibiting microbial growth, and nutraceutical delivery to make them more suitable for the pharmaceutical, cosmetic, nutraceutical, and food industries. Moreover, the active substances extracted from plants can be introduced into polymer films as plasticizers, crosslinkers, and additives to improve their mechanical properties, increase the degradability and thermal insulation, extend their shelf life, and enhance the safety and sensory quality of the packed products [26,27]. Particular attention should be paid to the natural bioactive components present in agro-industrial wastes. The valorization of these compounds with antioxidant and antimicrobial activities extracted from agricultural by-products can reduce the waste from the agricultural industries and transform them into value-added components that can be added to other industrial products and packaging materials [28]. Recently, various researchers [29,30,31,32] have reported the effectiveness of bioactive components with antioxidant and antimicrobial activities recovered from different agrifood wastes, such as durian leaf waste, distillers’ grain waste, grape marc and leaves and the waste parts from red cabbage, and olive pomace added to gelatine–, alginate–, and starch–chitosan–polyvinyl alcohol-based active packaging films.

Additionally, the enhanced recovery of bioactive compounds from wild-growing and widely cultivated medicinal plants is essential for promoting the sustainable agricultural practices that respond to environmental and economic challenges. For this reason, the escalating demand for natural and plant-based ingredients with antioxidant and antimicrobial properties extracted from herbs to fabricate active food packaging has been found. The effects of the active compounds extracted from Chinese traditional medicine herbs (*Polygonatum cyrtonema*), other Asian herbs (sappan, cinnamon), and Mediterranean herbs (oregano, thyme, rosemary, clove, satureja, lavender, garlic) on the antioxidant, antimicrobial, physical, and structural properties of different polymeric matrices (carboxymethyl cellulose,xanthan gum, flaxseed gum, pectin–gelatine, polypropylene, milk and soya proteins, carrageenan, chitosan, cellulose, alginate, resins, starch) were investigated [33,34].

Interestingly, in our recent studies [35], we showed that among some agro-industrial waste extracts and herb extracts, chokeberry pomace (ChP) and lemon balm (LB) exhibited the highest antioxidant features. 

It is known that black chokeberry (*Aronia melanocarpa* L.) has grown naturally in North America. Since the twentieth century, this plant has grown in Europe [36]. It is a food plant with fruits for producing juices, jams, tinctures, jellies, fruit teas, and dietary supplements. Chokeberries are rich in polyphenolic compounds, e.g., anthocyanins, proanthocyanidins, flavonols, flavanols, and phenolic acids. Due to the high content of polyphenolic compounds, these fruits have a high antioxidant potential. Their profound dark color results from the presence of anthocyanins, but the intensity depends on the anthocyanin aglycons [36,37]. Interestingly, chokeberry pomace (ChP), the main by-product of the agro-food industry, is still a rich source of natural colorants and phenolic compounds with antioxidant and antimicrobial properties. Therefore, the bioactive compounds present in ChP can benefit human health and should be recovered and applied in food, cosmetics, pharmaceuticals, and other industries [38].

Nevertheless, lemon balm (*Melissa officinalis* L) is a native plant in the Mediterranean area and Western Asia, but now it is cultivated worldwide. The different fresh or dried parts of this medicinal plant (leaves, flowers, and branches) contain various active ingredients, such as terpenoids, flavonoids, phenolic acids, tannins, and essential oils, having a fresh lemon odor and light yellow color. Importantly, phenolic acids (caffeic, cumaric, chlorogenic, ferulic, and rosmarinic acids) and flavonoids (apigenin, cynaroside, daidzein, kaempferol, quercetin, and rutin) are the crucial bioactive constituents of lemon balm, responsible for its antioxidant activities [39,40].

However, to the best of our knowledge, there are no references to the simultaneous use of DESs as “green” solvents for extracting the antioxidants from chokeberry pomace (ChP) and lemon balm (LB) and their plasticizing impact on alginate films.

Therefore, this research aimed to produce alginate films containing ChP and LB extracts as the potential applications for food packaging. Such films possess antioxidant properties due to the active compounds present in the extracts recovered from ChP agricultural by-products and LB medicinal plants. DESs were used to acquire the plant extracts, avoiding using harmful organic solvents. Moreover, the alginates that originate from brown algae are non-toxic and biodegradable. Thus, active and eco-friendly alginate films were obtained based on these components. We believe that experiments comparing how the extracts in different DESs influence the biopolymer films’ antioxidative, mechanical, and color properties have not been conducted previously. Moreover, chemometric tools were used to classify the alginate films loaded with ChP and LB extracts fabricated using different DESs and check the possible relationships between their antioxidant and mechanical properties and color parameters.

## 2. Materials and Methods

### 2.1. Materials

The information on some chemical compounds and plants, preparation of DESs, and method of active substance extraction from plants is contained in the previous publication [35].

For film preparations, sodium alginate (ALG) was obtained from Büchi Labortechnik AG (Flawil, Switzerland), and its average viscosity molecular weight was determined in our laboratory and was equal to 55,800 (K = 0.0178 mL/g and a = 1) [41]. Glycerol (Gly), purity of 99.5%, was bought from Avantor Performance Materials Poland S.A. (Gliwice, Poland).

### 2.2. Film Preparation

First, a 2% (*m*/*v*) sodium alginate solution was prepared, adding 1.5% (*m*/*v*) of glycerol. Next, 1.5 mL (5% (*v*/*v*)) of plant extract in a DES was added to 30 mL of alginate solution. In the case of an over-viscous DES extract, the extract was heated previously. The solutions were stirred for 15 min at ambient temperature. Then, the solutions were sonicated for two minutes in an ultrasonic bath to remove air bubbles. The final solutions were poured into circular dishes and left to dry at room temperature for 48–72 h. The dried films were removed from the dishes and were ready for testing.

### 2.3. Films’ Characterization

The AC of the prepared alginate films incorporating ChP and LB extracts based on five different DESs was determined by using the direct Quick, Easy, New, CHEap, and Reproducible procedure of quenching the 2,2-diphenyl-1-picrylhydrazyl (DPPH) radical reagent and the color formation of the CUPric Reducing Antioxidant Capacity (CUPRAC) reagent. The QUENCHER_DPPH_ and QUENCHER_CUPRAC_ assays were applied as described in our previous article [42] with some modifications. The AC results were expressed as μmol Trolox equivalents (TE) per 100 g of film sample. 

Calibration curves were prepared using TE working solutions in methanol between 2.00 × 10^−2^–1.00 × 10^−1^ μmol/mL and 6.00 × 10^−3^–7.00 × 10^−2^ μmol/mL for the QUENCHER_DPPH_ and QUENCHER_CUPRAC_ methods, respectively. Three calibration curves were plotted on the same day using the least squares method, resulting in the equations: %DPPH_scavenging_ = (702.89 ± 7.34) × c_TE_ − (4.61 ± 0.92) and A_450_ = (18.35 ± 0.08) × c_TE_ + (0.013 ± 0.001) with the determination coefficients, R^2^ = 0.9799 and 0.9979, respectively.

According to the QUENCHER_DPPH_ assay, in a test tube, 0.1 g of each powdered film sample and 6 mL of DPPH solution (60.8 μmol/L) were transferred and shaken vigorously using the Classic Vortex Mixer (Velp Scientifica Srl, Usmate (MB), Italy) for ten minutes to facilitate the reaction with the DPPH radical. After 15 min, the absorbance of the optically clear supernatant was measured at 517 nm using the Hitachi U-2900 spectrophotometer (Tokyo, Japan). The percentage of DPPH free radical scavenging activity was calculated by the following Equation (1):

DPPH scavenging activity (%) = [(Abs_blank_ − Abs_sample_)/Abs_blank_] × 100 (1)

where Abs_blank_ is the absorbance of the control DPPH solution without the film sample, and Abs_sample_ is the absorbance of the DPPH solution in the presence of the sample (powdered films or standard TE solutions).

In the QUENCHER_CUPRAC_ assay, in a test tube, 0.1 g of each powdered film sample was weighed, and 2 mL of CuCl_2_, 2 mL of ammonium acetate buffer, 2 mL of neocuproine, 3 mL of ethanol, and 1 mL of redistilled water were introduced. The obtained suspensions were vortexed for 10 min to facilitate the reaction with the CUPRAC reagent and incubated at room temperature in the dark for 20 min. The absorbance of the optically clear supernatants was measured spectrophotometrically at 450 nm.

The mechanical properties of the samples were studied using the testing machine, EZ-Test SX Texture Analyzer (Shimadzu, Kyoto, Japan). A paddle-shaped sample was stretched until rupture with a 10 mm/min stretching speed. From the stress–strain curve, the Young’s modulus (E), the stress at break, which can be named the tensile strength (σ), and the strain at break (ε) were determined. For all the samples, the stress at break was equal to the maximal stress, except for the films with B:CitA, for which the maximal stress was presented. The stress–strain curves of these samples had slightly different shapes, but to compare them better, the maximal stress was taken. Trapezium X software version 1.4.5 (Shimadzu, Kyoto, Japan) was applied to calculate the parameters mentioned. 

The film color was checked with the help of the Skin-Colorimeter CL 400 (Courage & Khazaka, Köln, Germany). The parameters L*, a*, b* of the CIE Lab trichromatic color model were determined, where: L*—brightness (from black (L* = 0) to white (L* = 100), a*—chromaticity parameter (form red (+a) to green (−a)), and b*—chromaticity parameter (from yellow (+b) to blue (•b)). Based on these parameters, the total color difference (ΔE) was calculated using the following Equation (2):

ΔE* = [(ΔL*)^2^ + (Δa*)^2^ + (Δb*)^2^]^1/2^ (2)

where ΔL* is the difference in brightness between a sample and the reference (L* • L_0_*), and Δa* and Δb* are the differences in these parameters between a sample and the reference (film of sodium alginate with glycerol). A white sheet of paper served as a test substrate. The color parameters for the white sheet of paper were as follows: L* = 91.07± 0.48; a* = 0.86 ± 0.11; b* = −1.94 ± 0.62.

### 2.4. Statistical and Chemometric Analyses

The presented results are average values with standard deviation (SD). The data were tested using one-way analysis of variance (ANOVA) and Duncan’s post hoc test at a significance level of 0.05. In addition, chemometric analyses as principal component analysis (PCA) and hierarchical cluster analysis (HCA) were conducted. PCA was employed to study the clustering and differentiation of 11 alginate films with two plant extracts obtained in five various DESs based on their antioxidant potential (QUENCHER_DPPH_ and QUENCHER_CUPRAC_), mechanical properties (E, σ, ε), and color measurements (L*, a*, b*). For visual analysis purposes, three-dimensional projections were generated where the axes, known as principal components (PCs) with high eigenvalues, represent the studied samples and variables. Each principal component (PC) is a linear combination of the original responses, and these PCs are orthogonal to one another. The PCA results were depicted on the three-dimensional graphs, considering the analyzed scores (different alginate films) and variables (antioxidant, mechanical and color parameters).

Moreover, the clustering method, namely hierarchical cluster analysis (HCA) with Ward’s method using Euclidean distances, was used to explore the arrangement of the investigated film samples and their evaluated properties into groups and depict the hierarchical relationships both within and between these groups. The HCA results were presented in two-dimensional dendrograms, which are plots illustrating the organizational structure of the studied samples and variables in a treelike format. 

Apart from PCA and HCA, the correlation matrix (Pearson’s correlation coefficients) was calculated to estimate the degree of linear association between the studied films’ parameters. 

All the statistical and chemometric analyses were conducted using the Statistical Windows software package (version 8.0, StatSoft, Tulsa, OK, USA).

## 3. Results and Discussion

### 3.1. Antioxidant Activity Results

**Figure 1:**
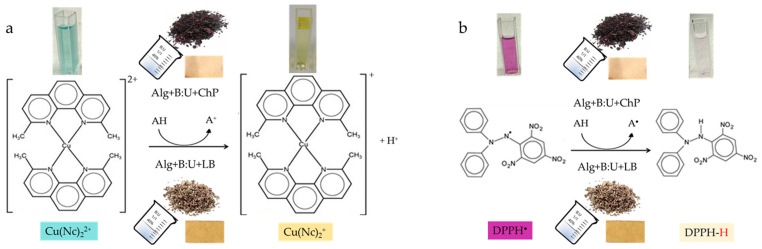
QUENCHER_CUPRAC_ (**a**) and QUENCHER_DPPH_ (**b**) mechanisms of antioxidant capacity measurements of alginate films loaded with chokeberry pomace (ChP) and lemon balm (LB).

The direct QUENCHER approach makes it possible to evaluate the antioxidant properties of the prepared films without preliminary antioxidant extraction from their insoluble matrix, taking advantage of the surface reaction phenomenon between bound antioxidants in solid film material and a liquid DPPH radical or CUPRAC reagent. The QUENCHER_CUPRAC_ method is only helpful in measuring the content of antioxidants with a single electron transfer (SET) mechanism, while the QUENCHER_DPPH_ assay can further quantify the antioxidants in a solid matrix with a hydrogen atom transfer (HAT) mechanism [43] (Figure 1).

The QUENCHER_DPPH_ and QUENCHER_CUPRAC_ values of the prepared alginate films without and with (ChP) or (LB) extracts based on five different DESs are listed in Table 1. 

The Duncan test indicated that the antioxidant potential of the enriched alginate films determined by the two modified analytical procedures differs significantly. It is noteworthy that the DPPH radical scavenging activity of the compounds bonded to the insoluble film matrix measured by using the direct spectrophotometric QUENCHER_DPPH_ procedure (348.3 ± 16.4–1174.9 ± 6.5 μmol TE/100 g) was higher than the reducing capacity of the copper(II)–neocuproine (Cu(II)-Nc) reagent (QUENCHER_CUPRAC_ = 33.7 ± 0.4–377.1 ± 6.5 μmol TE/100 g).

This suggests that the radical scavenging activity of the obtained films measured via the QUENCHER_DPPH_ methodology is not directly related to their reducing capacity analyzed via the QUENCHER_CUPRAC_ assay. The higher efficiency of the QUENCHER_DPPH_ approach in solubilizing and oxidizing antioxidants than the QUENCHER_CUPRAC_ can be explained by the bioactive compounds present in enriched alginate films having a higher ability to scavenge the DPPH radical than a reduction of Cu(II)-Nc to the colored Cu(I)-Nc chelate.

Moreover, these discrepancies between the AC results of alginate films loaded with ChP and LB extracts prepared by using the five types of DESs can be attributed to the various abilities of these DESs to extract secondary metabolites (phenolic acids, flavonoids, coumarins, carotenoids, terpenes, glucosinolates, and alkaloids) with an antioxidant effect naturally occurring in ChP and LB as well as the methodological differences in the applied analytical methods. ChP is a rich source of polyphenols such as procyanidins, anthocyanidins, and phenolic acids (chlorogenic and neochlorogenic acids, protocatechuic acid, rosmarinic acid), while flavanols (mainly quercetin and its derivatives) are present in low amounts [44].

On the contrary, flavonoids are important bioactive compounds of LB, among which are rutin, cynaroside, apigenin, daidzein, kaempferol, quercetin, isoquercetin, myricetin, luteolin, quercetrol, and hyperoside [39]. Moreover, rosmarinic, caffeic, chlorogenic, gentisic, and ferulic acids are dominant phenolic acids for the antioxidant activity of LB.

On the other hand, the application of DESs for the extractions of the antioxidant compounds from ChP and LB can lead to the breakdown of their high molecules into smaller ones with more phenolic hydroxyl and methoxy groups. Probably, these groups contributed significantly to the AC increase because they provide many hydrogen atoms that bond with DPPH^•^ radicals during radical scavenging or reduce Cu^2+^ to Cu^+^ by donating an electron in the case of the QUENCHER_CUPRAC_ assay (Figure 1).

Interestingly, alginate films incorporated with LB extracts prepared by using the five different DESs revealed a higher antioxidant potential (QUENCHER_DPPH_ = 469.0 ± 11.1–1174.9 ± 21.4 μmolTE/100 g and QUENCHER_CUPRAC_ = 162.6 ± 3.9–377.1 ± 6.5 μmolTE/100 g) than those loaded with ChP extracts (QUENCHER_DPPH_ = 348.3 ± 16.4–819.0 ± 32.1 μmolTE/100 g and QUENCHER_CUPRAC_ = 33.7 ± 0.4–252.1 ± 3.8 μmolTE/100 g). One possible explanation for these results is the fact that DES-based extracts of LB (DPPH = 3256–17,026 μmol TE/100 g and CUPRAC = 5925–42,985 μmol TE/100 g) are richer sources of antioxidants in comparison with ChP extracts (DPPH = 2921–14,833 μmol TE/100 g and CUPRAC = 4339–33,949 μmol TE/100 g), which was reported in our previous article [35]. However, all the DES-based extracts of LB and ChP had higher CUPRAC values than in the DPPH results. These results indicate that the binding of antioxidant compounds with the alginate film matrix differed. Therefore, the release of antioxidants that reduced the Cu(II)-Nc complex was hindered regardless of the DES used.

As can be seen, the viscosity and polarity of the chosen DES type to extract the antioxidants, depending on its constituent and the molar ratio of HBA to HBD, significantly affected the antioxidant properties of the prepared alginate films incorporating those extracts (Table 1).

The alginate films after the addition of LB extracts prepared based on B:CitA (1:1) and ChCl:CitA (1:1) revealed the highest AC via QUENCHER_DPPH_ (1174.9 ± 21.4 μmol TE/100 g) and QUENCHER_CUPRAC_ (377.1 ± 6.5 μmol TE/100 g), respectively. However, the lowest antioxidant potential (348.3 ± 16.4 and 33.7 ± 0.4 μmol TE/100 g) was determined by using these two analytical methods for the alginate films loaded with ChP extracts obtained using ChCl:CitA (1:1) and Glu:CitA (1:1), respectively.

Unexpectedly, the QUENCHER_DPPH_ and QUENCHER_CUPRAC_ values for the alginate films incorporated with LB and ChP extracts based on B:U (1:1) did not differ significantly (Duncan test, Table 1). Additionally, insignificant differences in QUENCHER_DPPH_ results for Alg+Glu:U+ChP, Alg+Glu:U+LB, Alg+Glu:CitA+ChP, and Alg+ChCl:CitA+LB were observed.

For comparison, the films based on polyvinyl alcohol (PVA)/cellulose nanocrystal (CNC) containing DES (ChCl:Glu = 2:1) and incorporating red grape skin had significantly higher DPPH (by 240.1%) compared to the pure PVA film [17]. Similarly, chitosan film loaded with astaxanthin extracted from shrimp wastes using a DES based on ChCl and lactic acid (LA) (1:2) clearly showed a high antioxidant potential determined using DPPH (5–35%) and ABTS (10–50%) depending on the astaxanthin concentration present in a polymeric matrix [18]. On the contrary, insignificant differences in the ABTS (2.2–2.9 mg TE/g) and DPPH (about 3.0 mg TE/g) values between neat pectin film, pectin film plasticized with glycerol, and pectin film plasticized with a ChCl:Gly-based natural DES and enriched with a DES extract of lavender [16]. Moreover, anthocyanins extracted from the Chilean *Luma chequen* (Molina) with different DESs (La:Gly (1:2), tartaric acid Ta:Gly (1:4), La:Glu (8:1), Gly:Glu (8:1), and ChCl:Gly (4:6)) and added to the matrix of edible carrageenan films did not significantly affect their antioxidant properties studied via the percentage of inhibition of DPPH (7.26–10.81%) and ABTS (20.87–30.50%) radicals (DPPH = 7.35% and ABTS = 21.72% for the control film) [19].

### 3.2. Mechanical Properties Test Results

Good mechanical properties aim to keep the film packaging intact while being transported and stored to protect the product inside it. The neat alginate film is so brittle that making mechanical measurements is difficult. Therefore, glycerol as a plasticizer was added to the biopolymer film. Moreover, glycerol can be a component of a DES and act as an HBD [8]. The obtained results are comparable because the amount of this compound is the same in all the samples. 

Table 2 encompasses the results of the mechanical properties tests. The alginate film had the highest Young’s modulus (198.31 ± 18.13 MPa) and tensile strength (17.15 ± 1.99 MPa) values and a low strain at break (30.26 ± 2.88%) value, which meant that this sample was stiffest, which resulted from the strong inter- and intramolecular interactions between macromolecules. 

The added plant extracts in DESs significantly influenced the mechanical properties of the biopolymer film. Generally, the Young’s modulus and maximal stress decreased, and the strain at break increased substantially in all the samples, indicating an improved film flexibility due to the weakening of the interactions between the macromolecules in the mixtures and the increasing free space in the polymer [45]. Only films with B:CitA (1:1) differed from all the films because all the measured parameters for these samples decreased. The reduction in the Young’s modulus was the smallest, indicating the fragility of the sample; the reduction in the tensile strength was significant, indicating the low tensile strength of the sample, and the elongation at break was about 5%, i.e., six times smaller than for the alginate film. Such reduction in the ability to deform and the high Young’s modulus indicated strong interactions between the components in the mixtures, suggesting the formation of a physical crosslinked network. As an HBD, citric acid could form hydrogen bonds with carboxylate ions in betaine and alginate, forming a crosslinked structure. Betaine, as a zwitterion with positive and negative charges, could not interact with each other owing to the shielding of the positive charge by methyl groups; its interactions with citric acid were via a carboxylate anion [46]. Thus, the mechanical properties of these films deteriorated.

The plasticizing effect of the added DESs depended on the structure of the DES components. The films with ChCl:CitA (1:1) were more flexible than the control and films with B:CitA, which was indicated by the lower Young’s modulus value and higher elongation value of the films with ChCl:CitA (1:1). The HBD compound was the same in both cases. Thus, HBA substances were responsible for the difference in the mechanical properties between both types of films. Citric acid interacted with choline chloride via a chlorine atom, while with betaine via a carboxylate anion. Moreover, CitA could also interact with the carboxylate groups of alginate in both cases.

On the other hand, the interactions of ChCl molecules with each other could be possible, in contrast to B, which could not interact with their molecules [46]. The differences resulted in different films’ properties, suggesting weaker interactions in films when ChCl:CitA (1:1) molecules were present. When analyzing the influence of the added extracts in the DESs on the mechanical properties, one should remember that the solution comprises sodium alginate, a polyanion (negatively charged), and glycerol, which could act as an HBD. Pontillo et al. [45] studied the impact of NaDESs B:LA and ChCl:LA on the burst strength (BS) and the distance at burst (DB) of chitosan films with these DESs. Both tested DESs revealed the plasticizing effect on chitosan film. Moreover, chitosan films with B:LA were significantly more flexible than those with ChCl:LA. It was the opposite in our case, probably because of the different interactions in the mixtures resulting from different biopolymers, namely chitosan is a polycation and alginate is a polyanion. 

Alginate with Glu:U (1:1) formed the most flexible films, indicating the weakest interactions in this system. Flexible and neutral glucose molecules and small urea molecules probably facilitate polymeric chain movements, creating free volume in the polymer. 

The type of plant extract only slightly affected the mechanical properties of the films. The presence of LB extract in the DESs in the alginate films resulted in a slight increase in the strain at break and a slight decrease in the Young’s modulus and maximal stress, indicating a better flexibility of films with LB than with a ChP extract. The substances in extracts contributed to the overall interactions in the films as well. 

The thickness of the alginate film plasticized with glycerol was 0.1012 ± 0.0046 mm. The presence of extracts in the DESs in the films resulted in significant thickness increases (Table 2). This phenomenon depended both on the DES and the extract type. Generally, alginate with a ChP extract formed thicker films than LB extract due to the different ingredients of the extracts. 

The thickest films were obtained for alginate with B:CitA (1:1) (0.2692 ± 0.0062 mm (ChP) and 0.2402 ± 0.0036 mm (LB)) and ChCl:CitA (1:1) (0.2711 ± 0.0087 mm (ChP) and 0.2517 ± 0.0088 mm (LB)). This behavior can be explained by the chemical structure and the sizes of molecules forming the DESs. These samples were also characterized by higher Young’s modulus values and lower elongations at breaks than the remaining ones. The introduced DES molecules interacted with the biopolymer functional groups via hydrogen bonds and electrostatic interactions [47], which resulted in a higher thickness of these films.

In contrast, alginate with extracts in Glu:U formed the thinnest films, which resulted from the structure of both the DES components and the small size of urea molecules. It should also be emphasized that glycerol and water molecules could interact in all the samples.

Also, the ATR−FTIR spectra of the films strongly depend on the DES type. The spectra of the films with extracts in the DESs are presented in the Supplementary Materials.

### 3.3. Color Measurement Results

**Figure 2:**
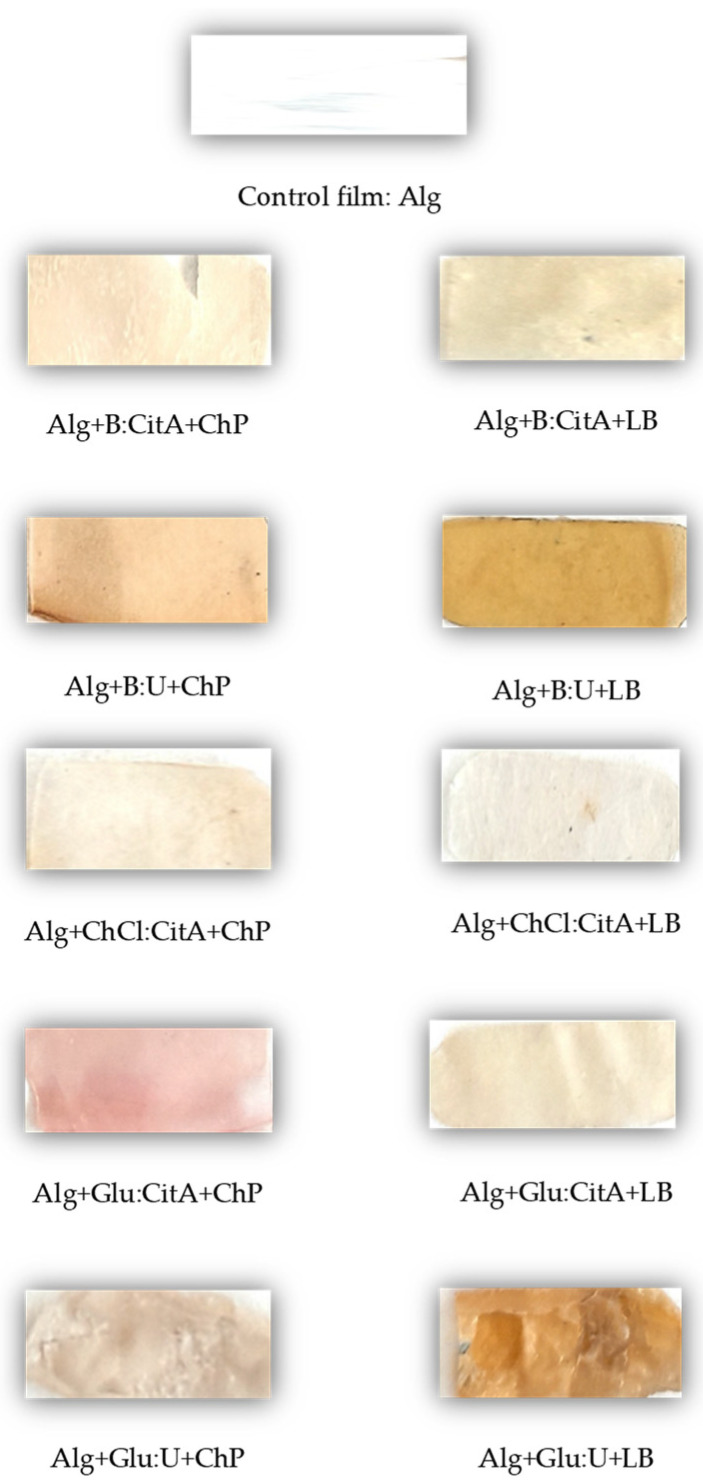
Appearance of the prepared alginate films.

A film’s color is a vital feature of film packaging because it affects how consumers perceive the product. The values of color parameters (L*, a*, and b*) and the total color difference (ΔE) of the prepared films are included in Table 3. Plasticized alginate film was colorless and transparent. Its lightness value was equal to 92.68, and a* and b* values were 0.62 and ‒1.75, respectively. The plant extracts in the different DESs added to the polymeric films made the films colorful, reflected in the change in the parameters L*, a*, and b*. Generally, the prepared alginate films with a chokeberry pomace extract were mainly shades of beige or pink, whereas the samples with a lemon balm extract were beige or brown (Figure 2). 

The lightness parameter, L*, shows how bright the sample is, and this parameter depended mainly on the DES type. The lightness of films with plant extracts in ChCl:CitA (1:1) was similar to that of the film without an extract. In contrast, the films with extracts in Glu:U (1:1) had the least L* values, indicating the highest amount of gray shades contributing to the film’s color. 

The values of the a* and b* parameters depended on the extract type and DES; the a* values were higher for all samples with ChP extracts than those with LB extracts, indicating the higher contribution of red/pink to the film color for the samples with ChP extracts. In contrast, the b* values were lower for the samples with chokeberry pomace extracts than those with lemon balm extracts, which indicated a higher contribution of yellow/brown in film color of the films with lemon balm. The highest a* value (16.10 ± 0.73) and the lowest b* value (−1.72 ± 0.22) were found for the alginate film with ChP in Glu:CitA (1:1), and this film was pink. Other distinguishing values were the highest b* (23.28 ± 0.62) and the lowest a* (−1.04 ± 0.60) values for the sample with LB in B:U (1:1), and this film had the most intense brown color. The slightly less brown film was with LB in Glu:U (1:1), for which b* was equal to 19.31 ± 2.54) and a* = 1.75 ± 1.13. 

The colors of the samples were given by substances containing chromophoric groups, such as carbonyl groups, aromatic rings, and conjugated double bonds. These substances were extracted from plants using the different DESs, which indicates that depending on the DES used, extracts with varying compositions of active compounds were obtained, which was also demonstrated by other results of tests on the antioxidant properties of the films.

Other researchers applied the different DESs to extract the carotenoids from orange peels [47]. The results revealed the highest efficiency of carotenoid extraction in hexane and octanoic acid mixed with L-Proline (C8:Pro) due to a potential interaction between these compounds. Moreover, the extracts in the different DESs exhibited a higher AC than in hexane, sunflower oil, and olive oil, which was explained by the inherent antioxidant activity of the DESs and the extraction of other antioxidants, such as tocopherols. The color measurements of the extracts in the different DESs, hexane, octanoic acid, sunflower oil, and olive oil showed the highest a* and b* values for the extracts in hexane and in C8:Pro, indicating the relation between the a* and b* values and the carotenoid content, which was most remarkable in these two extracts.

### 3.4. Multivariate Analysis

#### 3.4.1. Principal Component Analysis

The PCA was applied to compare and classify the alginate films loaded with ChP and LB extracts based on the different types of DESs according to their antioxidant and mechanical properties and color parameters. The first three PCs, namely PC1, PC2, and PC3, were selected as significant (Kaiser’s rule) because their eigenvalues were larger than 1 (3.62, 2.14, and 1.48) and accounted for 80.51% of the variability in the dataset. However, the remaining six generated PCs yielded progressively lower eigenvalues (<1; 0.96, 0.37, 0.23, 0.19, 0.0065, and 0.0024, respectively) and did not explain the variability in the data (<19.50% total). The PC1 positively correlated with b* (0.7742), QUENCHER_DPPH_ (0.6733), and ε (0.5746), while negatively with σ (−0.9266), E (−0.7472), and L* (−0.7117). The PC2 inversely correlated with QUENCHER_CUPRAC_ (−0.7030), thickness (−0.6712), and L* (−0.5330) but positively with ε (0.6825). At the same time, PC3 was highly contributed by a* (0.8413) and thickness (0.5894).

**Figure 3:**
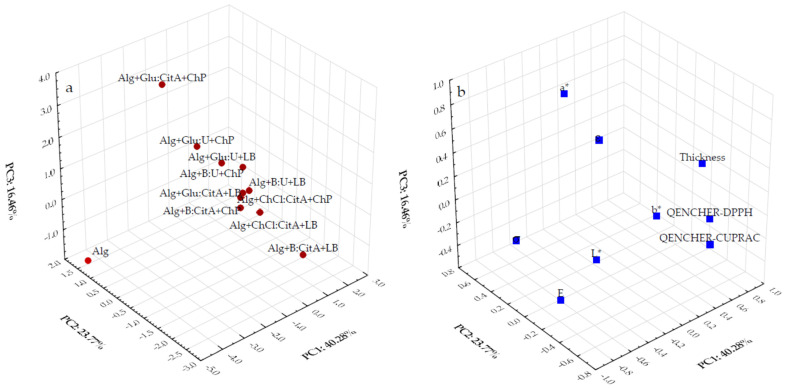
Three-dimensional principal component analysis plots based on (**a**) the scores (eleven fabricated alginate films) and (**b**) variables (L*, a*, b*, E, σ, ε, thickness, QENCHER_DPPH_, QUENCHER_CUPRAC_).

A visual design of the first three PCs is depicted on the PCA scores 3D plot (Figure 3a) of the film samples and loading 3D plot (Figure 3b).

The 3D plot of scores (Figure 3a) indicates that the studied alginate films fell into five distinct groups. Five films incorporating ChP and LB extracts in the DESs based on ChCl:CitA, Alg+Glu:CitA+LB, Alg+B:CitA+ChP, and Alg+B:U+LB created distinct clusters. This group generally had a similar moderate antioxidant potential (QENCHER_DPPH_ = 348.3–901.2 μmol TE/100 g, QUENCHER_CUPRAC_ = 90.7–377.1 μmol TE/100 g), and high thickness (0.2064–0.2711 mm) and b* (−0.60–23.28) values. The Alg+B:CitA+LB film with the highest antioxidant properties (QENCHER_DPPH_ = 1174.9 μmol TE/100 g, QUENCHER_CUPRAC_ = 322.6 μmol TE/100 g), the lowest strain at break (ε = 5.29%), and low redness (a* = −0.17) was moving away from this group. Moreover, three films enriched with ChP and LB extracts prepared using the DES containing Glu:U (1:1) and Alg+B:U+ChP having the high ε (77.31–122.42%) and a* (1.75–3.47) and low thickness (0.1742–0.2268 mm), E (0.39–2.55 MPa), and L* (72.39–82.19) were separated from the other studied film samples. The Alg+Glu-CitA+ChP was clearly discriminated from the fabricated films due to the highest redness (a* = 16.10) and the lowest ability to reduce Cu(II)-Nc complex (QUENCHER_CUPRAC_ = 33.7 μmol TE/100 g). Evidently, the control Alg sample with the longest distance from the other alginate films incorporating ChP and LB extracts based on the different DESs had the highest values of σ (17.15 MPa) and E (198.31 MPa) and the most lightness (L* = 92.68), whereas this biopolymer with less thickness (0.1012 mm) and the lowest yellowness (b* = −1.75) did not reveal an antioxidant potential.

As can be seen from the factor loading plot (Figure 3b), variables were grouped in three clusters, including (1) E, σ, and lightness (L*), (2) thickness, yellowness (b*), and antioxidant properties, and (3) ε and redness (a*).

#### 3.4.2. Hierarchical Cluster Analysis

**Figure 4:**
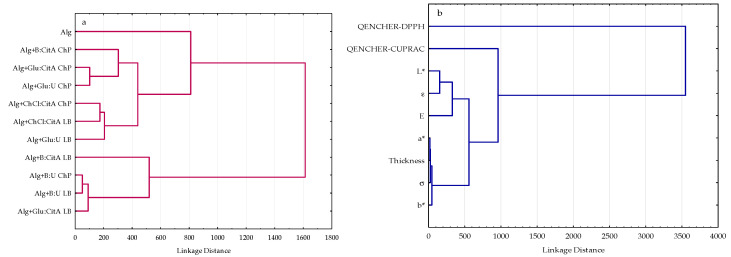
Dendrograms of hierarchical cluster analysis for (**a**) the fabricated alginate films and (**b**) the studied variables.

The HCA was applied to the visualization of the the similarity among 11 prepared alginate films based on nine variables (QENCHER_DPPH_, QUENCHER_CUPRAC_, L*, a*, b*, E, σ, ε, thickness). The generated dendrograms presented in Figure 4 indicate the similarity of the objects (film samples and variables) within the studied dataset as tree structures.

As can be seen, at a distance of 1600, the HCA separated the fabricated alginate films into two main clusters (Figure 4a). The first cluster included three inter-groups: (1) Alg+Glu:U+LB, Alg+ChCl:Cit+LB, and Alg+ChCl:Cit+ChP, (2) Alg+Glu:U+ChP, Alg+Glu:CitA+ChP, and Alg+B:Cit+ChP, and (3) the control film (Alg). It can be noted that the samples from the first inter-cluster had the lowest QUENCHER_DPPH_ (348.3–548.0 μmolTE/100 g) and the highest QUENCHER_CUPRAC_ (233.4–377.1 μmolTE/100 g) values and moderate redness (a* = 0.32–1.75). However, the second inter-cluster, consisting of the alginate films incorporating ChP extracts, had the highest redness (a* = 2.10–16.10), the lowest ability to reduce the Cu(II)-Nc complex (QUENCHER_CUPRAC_ = 33.7–92.6 μmol TE/100 g), and a low DPPH radical scavenging activity (QUENCHER_DPPH_ = 436.7–670.9 μmol TE/100 g). The most lightness (L* = 92.68) control Alg film without antioxidant properties and having the highest E (198.31 MPa) and σ (17.15 MPa) but the lowest thickness (0.1012 mm) and yellowness (b* = −1.75) created the separate inter-group. These HCA results confirmed the PCA results presented in Figure 3a. Moreover, the second cluster was divided into two inter-clusters: (1) Alg+Glu:CitA+LB, Alg+B:U+LB, and Alg+B:U+ChP, and (2) Alg+B:CitA+LB (Figure 4a). These three film samples were associated with moderate antioxidant properties (QENCHER_DPPH_ = 819.0–901.2 μmol TE/100 g, QUENCHER_CUPRAC_ = 162.6–207.9 μmol TE/100 g) and a similar L* (81.33–88.94) and thickness (0.2064–0.2268 mm). Nevertheless, Alg+B:CitA+LB was distanced from this inter-cluster because it had a high antioxidant potential QENCHER_DPPH_ = 1174.9 μmol TE/100 g, QUENCHER_CUPRAC_ = 322.6 μmol TE/100 g) and E (104.77 MPa), the lowest ε (5.29%), and was thicker (0.2402 mm).

Additionally, the HCA results indicate that the two antioxidative, three mechanical, and three color parameters as variables comprised the three main groups (Figure 4b). The dendrogram separated the QUENCHER_DPPH_ method from the other variables, allowing the determination of the lipophilic antioxidants present in the studied films by applying DPPH radical quenching via the HAT mechanism and direct reduction through the SET mechanism. Furthermore, the QUENCHER_CUPRAC_ assay measuring both hydrophilic and lipophilic antioxidants in the prepared alginate biopolymers formed one cluster. It is noteworthy that the third cluster was composed of two subgroups consisting of (1) L*, ε, E and (2) a*, b*, σ, thickness (Figure 4b). This clearly depicted that the added extracts based on the DESs affected the strain at break (ε), modulus of elasticity (E), tensile strength (σ), lightness (L*), and yellowness (b*). The less light alginate films had more yellowness (b*) and higher ε values. In addition, more yellow films revealed low σ values, while the σ results increased with increasing lightness (L*). However, the Young’s modulus (E) results of the enriched films were reduced when the strain at break (ε) values were enhanced.

#### 3.4.3. Correlation Analysis

**Figure 5:**
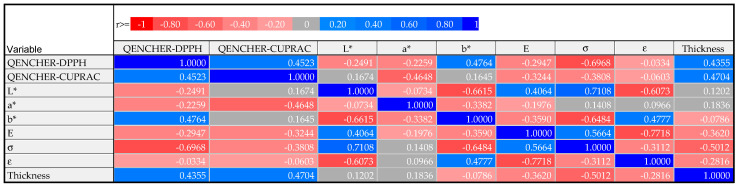
Correlation matrix.

The positive and negative correlations between the antioxidant, mechanical, and color parameters of the eleven alginate films without and with extracts prepared from ChP and LB based on the five various DESs are presented as a correlation matrix in Figure 5.

The calculated Pearson correlation coefficient (r = 0.7108, *p* = 0.014) confirmed the significant positive relationship between the lightness (L*) of the prepared films and their tensile strength (σ). This suggests that more light biopolymers loaded with ChP and LB extracts had higher tensile strength values. 

On the other hand, negative significant correlations were found between E − ε (r = −0.7718, *p* = 0.0054), DPPH − σ (r = −0.6968, *p* = 0.017), L* − b* (r = −0.6615, *p* = 0.027), b* − σ (r = −0.6484, *p* = 0.031), and L* − ε (r = −0.6073, *p* = 0.048). Evidently, the films with a higher stress at break had less yellowness, and they were weaker scavengers of DPPH radicals, whereas the strain at break (ε) substantially decreased with an increased Young’s modulus (E) and lightness (L*) of the studied alginate films. Moreover, the films with more yellow color revealed lower L* values.

## 4. Conclusions

The alginate films with chokeberry pomace (ChP) or lemon balm (LB) extracts prepared based on the five DESs such as betaine:citric acid (B:CitA), betaine:urea (B:U), choline chloride:citric acid (ChCl:CitA), glucose:citric acid (Gu:CitA), and glucose:urea (Gu:U) at a molar ratio of 1:1 were obtained via the casting method. ChP and LB extracts can be a source of antioxidant compounds, with DESs as green solvents, and simultaneously as plasticizers that can be used in food packaging. 

The films’ antioxidant capacity, mechanical properties, and color parameters were successfully determined. The conducted studies let us draw the following conclusions:

- The antioxidant capacity and color parameters (a* and b*) significantly depended on the extract and DES type;
- The antioxidant capacity of the films with LB was greater (QUENCHER_DPPH_ = 469.0–1174.9 μmol TE/100 g and QUENCHER_CUPRAC_ = 162.6–377.1 μmol TE/100 g) than that of the films with ChP (QUENCHER_DPPH_ = 348.3–819.0 μmol TE/100 g and QUENCHER_CUPRAC_ = 33.7–252.1 μmol TE/100 g), indicating a higher content of compound able to react with DPPH and Cu(II)-Nc complex in the LB extract;
- The added plant extracts based on the various DESs suggested interactions between alginate and extracts as well as the retention of antioxidant compounds in the film matrices;
- The mechanical properties and lightness (L*) depended on the DES type, while the extract type had a negligible influence on these values.

Multivariate analysis showed some interesting, clear correlations for these films, both positive and negative:

- The more that the light biopolymer films were loaded with plant extracts, the higher the value of the tensile strength;
- The higher the value of the Young’s modulus, the lower the value of the strain at break;
- The lower the value of the strain at break, the higher the lightness of the film;
- The higher the lightness, the lower the b* value and the lower the yellowness;
- The weaker the yellowness, the higher the tensile strength;
- The higher the tensile strength, the weaker the DPPH scavenger.

It is challenging to choose the best film composition because the films, Alg+ChCl:CitA+ChP, Alg+ChCl:CitA+LB, Alg+Glu:CitA+ChP, and Alg+Glu:CitA+LB, which had quite good mechanical properties and a suitable color (beige or light pink and transparent), had a low antioxidant potential, and contrarily, those with good antioxidant capacities had worse mechanical properties and a dark color.

The obtained results demonstrate that the alginate films loaded with ChP and LB extracts prepared using the five different DESs can be promising active biodegradable packaging for preserving the quality of products in the food, cosmetics, and pharmaceutical sectors as well.

There is a need for further research to determine the individual bioactive compounds in the added DES-based extracts and identify their role (as antioxidants, antimicrobials, antifungal agents, plasticizers, crosslinkers, etc.) and their interactions with other ingredients of the alginate film matrix to investigate their efficacy in improving the quality and sensory attributes of products along with their shelf life extension.

## Figures and Tables

**Table 1:** Antioxidant capacity of alginate films loaded with chokeberry pomace and lemon balm extracts based on five different deep eutectic solvents.

Sample	QUENCHER_DPPH_ ± SD (µmol TE/100 g)	QUENCHER_CUPRAC_ ± SD (µmol TE/100 g)
Alg	<DL	<DL
Alg+B:CitA+ChP	670.9 ± 41.7 ^e^	90.7 ± 0.4 ^b^
Alg+B:CitA+LB	1174.9 ± 21.4 ^h^	322.6 ± 14. 8 ^g^
Alg+B:U+ChP	819.0 ± 32.1 ^f^	163.3 ± 1.6 ^c^
Alg+B:U+LB	861.1 ± 57.5 ^f,g^	162.6 ± 3.9 ^c^
Alg+ChCl:CitA+ChP	348.3 ± 16.4 ^a^	252.1 ± 3.8 ^f^
Alg+ChCl:CitA+LB	469.0 ± 11.1 ^b,c^	377.1 ± 6.5 ^h^
Alg+Glu:CitA+ChP	436.7 ± 22.7 ^b^	33.7 ± 0.4 ^a^
Alg+Glu:CitA+LB	901.2 ± 20.2 ^g^	207.9 ± 5.0 ^d^
Alg+Glu:U+ChP	509.0 ± 11.2 ^c,d^	92.6 ± 1.3 ^b^
Alg+Glu:U+LB	548.0 ± 15.4 ^d^	233.4 ±8.4 ^e^

Values presented as mean from three replications ± standard deviation (SD); different letters within the same column indicate significant differences between antioxidant capacity of the studied alginate films determined by two analytical assays (one-way ANOVA and Duncan test, *p* < 0.05); Ch—chokeberry pomace, LB—lemon balm, B—betaine, Glu—glucose, CitA—citric acid, U—urea, ChCl—choline chloride.

**Table 2:** Mechanical properties: Young’s modulus, E [MPa]; maximal stress that the sample endures, σ_max_ [MPa]; strain at break ε [%] of the studied samples, and their thicknesses (mm).

Sample	E ± SD [MPa]	σ ± SD (MPa)	ε ± SD (%)	Thickness ± SD (mm)
Alg	198.31 ± 18.13 ^e^	17.15 ± 1.99 ^d^	30.26 ± 2.88 ^b^	0.1012 ± 0.0046 ^a^
Alg+B:CitA+ChP	122.59 ± 14.50 ^d^	1.87 ± 0.04 ^a^	5.85 ± 0.98 ^a^	0.2692 ± 0.0062 ^i^
Alg+B:CitA+LB	104.77 ± 36.71 ^c^	1.68 ± 0.11 ^a^	5.29 ± 1.07 ^a^	0.2402 ± 0.0036 ^g^
Alg+B:U+ChP	2.55 ± 0.19 ^a^	2.12 ± 0.42 ^a^	77.31 ± 8.62 ^d^	0.2268 ± 0.014 ^e,f^
Alg+B:U+LB	1.41 ± 0.12 ^a^	1.35 ± 0.09 ^a^	102.78 ± 1.75 ^e^	0.2064 ± 0.0141 ^d^
Alg+ChCl:CitA+ChP	23.25 ± 0.99 ^b^	5.61 ± 0.63 ^b^	48.39 ± 6.13 ^c^	0.2711 ± 0.0087 ^i^
Alg+ChCl:CitA+LB	18.52 ± 3.06 ^a,b^	6.61 ± 0.27 ^b,c^	56.92 ± 2.78^c^	0.2517 ± 0.0088 ^g,h^
Alg+Glu:CitA+ChP	7.62 ± 0.80 ^a,b^	7.86 ± 0.61 ^c^	75.54 ± 2.61 ^d^	0.2394 ± 0.0093 ^f,g,h^
Alg+Glu:CitA+LB	6.35 ± 0.42 ^a,b^	6.47 ± 1.68 ^b,c^	80.58 ± 11.93 ^d^	0.2149 ± 0.0066 ^d,e^
Alg+Glu:U+ChP	0.84 ± 0.07 ^a^	1.05 ± 0.33 ^a^	112.83 ± 20.26 ^e,f^	0.1877 ± 0.0135 ^c^
Alg+Glu:U+LB	0.39 ± 0.03^a^	0.89 ± 0.05^a^	122.42 ± 5.32 ^f^	0.1742 ± 0.0103 ^b^

Values presented as mean from five replications ± standard deviation (SD); the different letters within the same column indicate the significant differences between E, σ, ε, and the thickness results of the studied alginate films (one-way ANOVA and Duncan test, *p* < 0.05); Ch—chokeberry pomace, LB—lemon balm, B—betaine, Glu—glucose, CitA—citric acid, U—urea, ChCl—choline chloride.

**Table 3:** The studied samples’ color parameters (L*, a*, and b*) and total color difference (ΔE).

Sample	L* ± SD	a* ± SD	b* ± SD	ΔE
Alg	92.68 ± 1.55 ^g^	0.62 ± 0.60 ^c^	−1.75 ± 0.40 ^a^	
Alg+B:CitA+ChP	82.23 ± 2.37 ^c^	2.53 ± 0.23 ^f^	5.44 ± 0.36 ^e^	12.83
Alg+B:CitA+LB	86.32 ± 1.25 ^d^	−0.17 ± 0.10 ^b^	7.37 ± 0.60 ^f^	11.15
Alg+B:U+ChP	82.19 ± 1.58 ^c^	3.47 ± 0.46 ^g^	12.23 ± 0.76 ^g^	17.70
Alg+B:U+LB	81.33 ±1.12 ^c^	−1.04 ± 0.60 ^a^	23.28 ± 0.62 ^i^	27.19
Alg+ChCl:CitA+ChP	90.59 ± 0.70 ^f^	1.44 ± 0.45 ^d^	2.43 ± 0.24 ^c^	4.72
Alg+ChCl:CitA+LB	92.74 ± 0.87 ^g^	0.32 ± 0.19 ^b,c^	−0.60 ± 0.22 ^b^	1.19
Alg+Glu:CitA+ChP	85.24 ± 0.64 ^d^	16.10 ± 0.73 ^h^	−1.72 ± 0.22 ^a^	17.18
Alg+Glu:CitA+LB	88.94± 1.96 ^e^	0.24 ± 0.20 ^b,c^	2.85 ± 0.65 ^c^	5.94
Alg+Glu:U+ChP	75.43 ± 1.57 ^b^	2.10 ± 0.14 ^e,f^	4.17 ± 1.04 ^d^	18.29
Alg+Glu:U+LB	72.39 ± 1.64 ^a^	1.75 ± 1.13 ^d,e^	19.31 ± 2.54 ^h^	29.26

Values presented as mean from five replications ± standard deviation (SD); the different letters within the same column indicate the significant differences between L*, a*, b*, and the ΔE results of the studied alginate films (one-way ANOVA and Duncan test, *p* < 0.05); Ch—chokeberry pomace, LB—lemon balm, B—betaine, Glu—glucose, CitA—citric acid, U—urea, ChCl—choline chloride.

## Data Availability

Data are contained within the article.

## Supplementary Materials

The following supporting information can be downloaded at https://www.mdpi.com/article/10.3390/polym16142084/s1, Figure S1: ATR−FTIR spectra of the films: plasticized alginate, Alg+B:CitA+ChP and Alg+B:CitA+LB, Figure S2: ATR−FTIR spectra of the films: plasticized alginate, Alg+B:U+ChP and Alg+B:U+LB, Figure S3: ATR−FTIR spectra of the films: plasticized alginate, Alg+ChCl:CitA+ChP and Alg+ChCl:CitA+LB, Figure S4: ATR−FTIR spectra of the films: plasticized alginate, Alg+Glu:CitA+ChP and Alg+Glu:CitA+LB, Figure S5: ATR−FTIR spectra of the films: plasticized alginate, Alg+Glu:U+ChP and Alg+Glu:U+LB.
