# Supplementary material for: Influence of Different Deep Eutectic Solvents and Plant Extracts on Antioxidant, Mechanical, and Color Properties of Alginate Film
Source: Polymers (Basel). 2024 Jul 22;16(14):2084. doi: 10.3390/polym16142084 (PMC11280554; doi:10.3390/polym16142084)
Supplement: Supplementary file 1 [file polymers-16-02084-s001.zip › polymers-3105416-supplementary.pdf]

Supplementary Materials

Influence of different DESs and plant extracts on antioxidant, mechanical, and color properties of alginate film

Jolanta Kowalonek*[1], Malo Hamieau[2], Aleksandra Szydłowska-Czerniak[3]

[1] Department of Biomedical and Polymer Chemistry, Faculty of Chemistry, Nicolaus Copernicus University in Toruń, Gagarina 7, 87-100 Toruń, Poland; jolak@umk.pl
[2] Univ Rennes, IUT de Rennes, F-35000 Rennes, France; malo.hamieau@etudiant.univ-rennes1.fr
[3] Department of Analytical Chemistry and Applied Spectroscopy, Faculty of Chemistry, Nicolaus Copernicus University in Toruń, Gagarina 7, 87-100 Toruń, Poland; olasz@umk.pl
* Correspondence: jolak@umk.pl

1. Materials and Methods

1.1. Methods

Infrared spectra of the films were collected using the Nicolet iS5 (Thermo Fisher Scientific, Waltham, MA, USA) spectrophotometer equipped with ATR module (Pike Technologies, Inc. Madison, WI, USA) with built ZnSe crystal having 45° angle of incidence. The spectra were registered with 4 cm^{-1} resolution, 32 scans, and the range of wavenumbers 4000–550 cm^{-1}.

2. Results and Discussion

The ATR–FTIR spectroscopy provides information from about one μm of the sample. The film thickness was about 200 μm. Thus, the collected data was limited to very thin fragments of the films. Figures S1–S5 present ATR–FTIR spectra of the alginate films with different DESs and extracts. It is seen that DESs strongly affected the alginate spectrum, whereas the type of extracts had less influence on the shape of the polysaccharide spectrum. Figure S1 shows the ATR–FTIR film spectra of the plasticized alginate, Alg+B:CitA+ChP and Alg+B:CitA+LB. In the alginate spectrum, there are bands characteristic of this polysaccharide. A broad absorption band with a maximum of 3280 cm^{-1} was assigned to hydrogen-bonded O–H stretching modes. The maxima at 2935 cm^{-1} and 2886 cm^{-1} came from C–H asymmetric and symmetric stretching, respectively. The 1600 cm^{-1} and 1407 cm^{-1} bands indicated COO^{-} asymmetric and symmetric stretching, respectively. Next, a band at 1297 cm^{-1} was attributed to C–H deformation vibrations. The band with two maxima at 1084 cm^{-1} and 1026 cm^{-1} came from stretching vibrations of C–O–C in glycosidic linkages. The band at 947 cm^{-1} was associated with C–H bending vibrations. The band at 930 cm^{-1} was assigned to O–H deformation and 854 cm^{-1} to C–O symmetric stretching vibrations; both indicate glycerol presence. The band at 815 cm^{-1} was related to out-of-plane stretching of –C–H in polysaccharides.

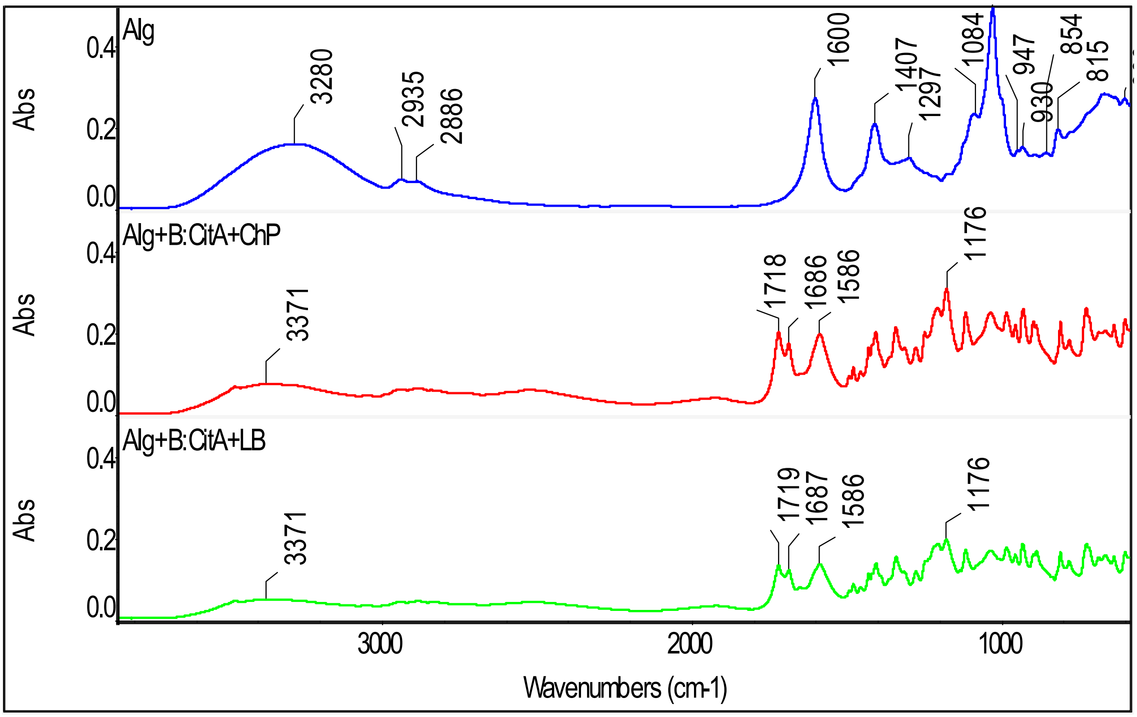

Figure S1. ATR−FTIR spectra of the films: plasticized alginate, Alg+B:CitA+ChP and Alg+B:CitA+LB.

The spectra of alginate films with extracts in DESs were formed due to the superposition of the spectra of the initial compounds. Several bands not present in the initial alginate are seen in all films with DESs. These bands are characteristics of DESs' ingredients. The absorption band characteristics of alginate overlapped with those from DESs. One can observe a broad absorption band in the 3000 cm^{-1} – 3600 cm^{-1} range. This band comes from hydrogen-bonded O-H and N-H (in the case of urea) groups. This band was shifted in higher wavenumbers, indicating changes in the film structure. A band at about 1720 cm^{-1} was assigned to C=O bond vibrations in the spectra of films containing citric acid (Fig. S1, S3 S4). A band at about 1600 cm^{-1} was assigned to a carbonyl band of carboxylate anion and at 1610 cm^{-1} to vibrations of C=O in urea. Also, band at about 1400 cm^{-1} was related to the vibrations of carboxylate ions in betaine and alginate.

Moreover, in the 950 cm^{-1} - 1100 cm^{-1} region, a band of C-O modes of alginate and DES' ingredients overlapped. The changes in maxima position in infrared spectra indicated interactions between blend components. Due to the several compounds in the systems and overlapping absorption bands, the analysis was complex.

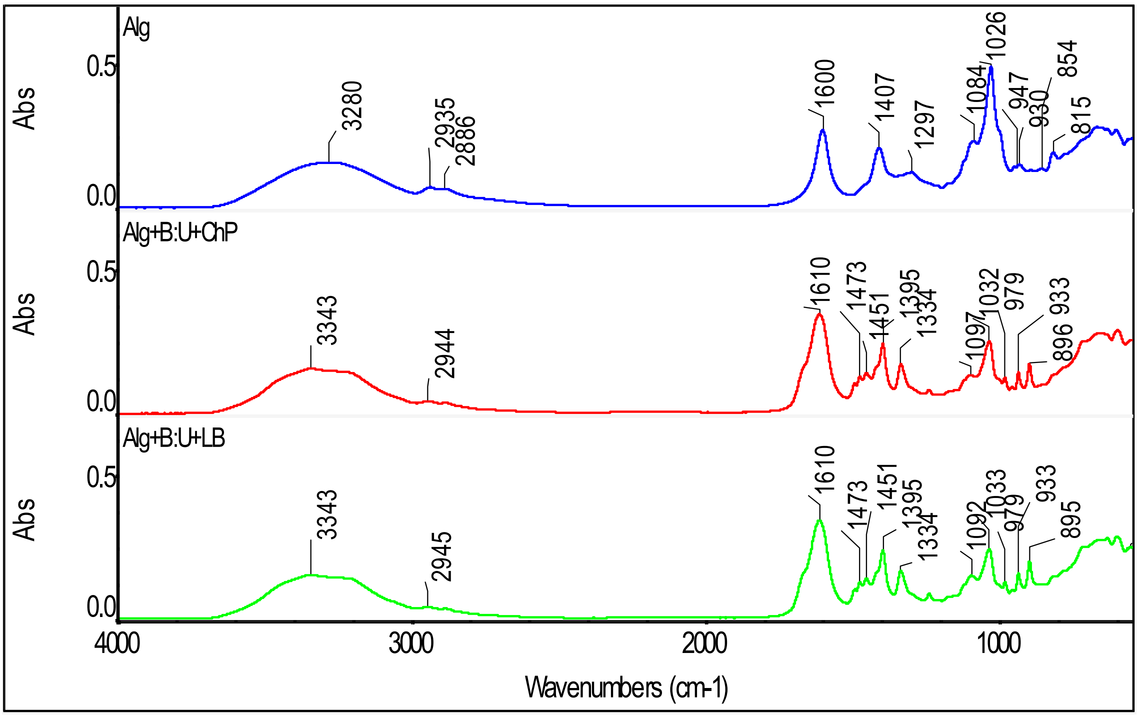

Figure S2. ATR−FTIR spectra of the films: plasticized alginate, Alg+B:U+ChP and Alg+B:U+LB.

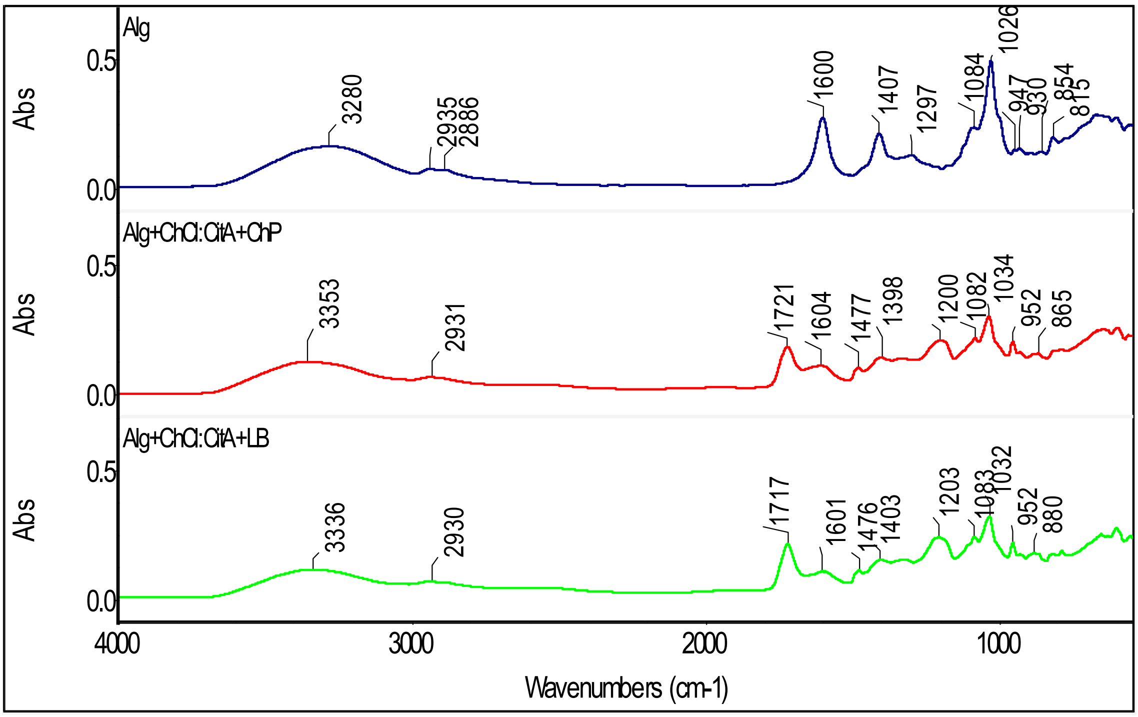

Figure S3. ATR−FTIR spectra of the films: plasticized alginate, Alg+ChCl:CitA+ChP and Alg+ChCl:CitA+LB.

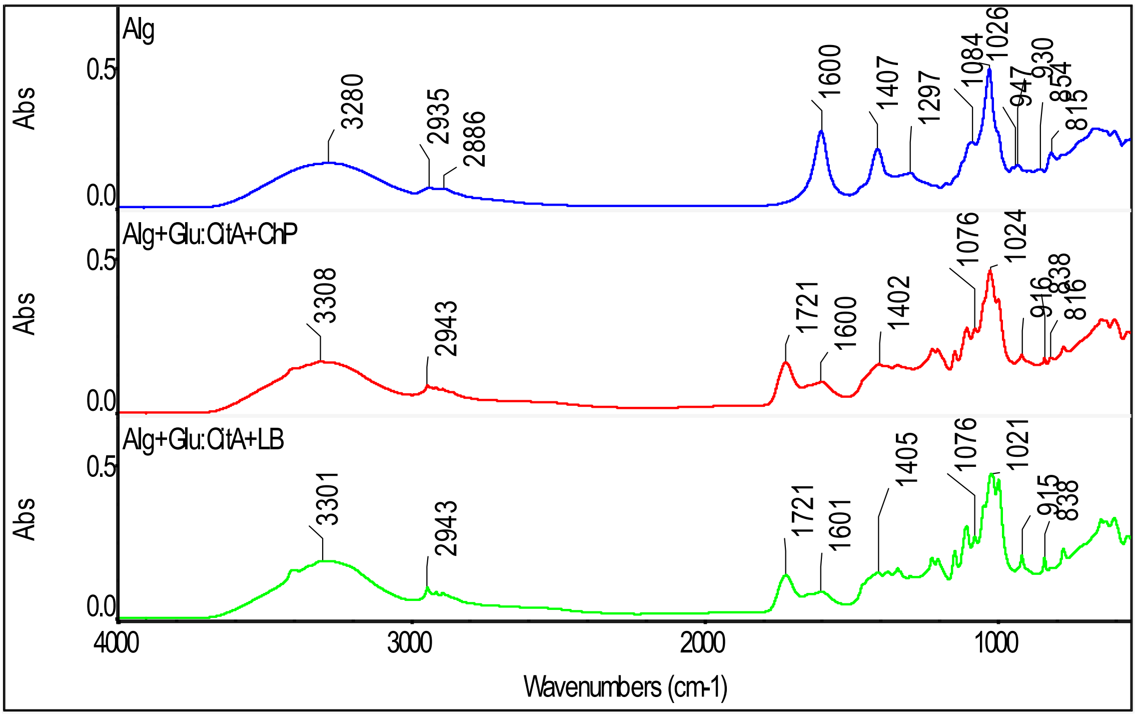

Figure S4. ATR−FTIR spectra of the films: plasticized alginate, Alg+Glu:CitA+ChP and Alg+Glu:CitA+LB.

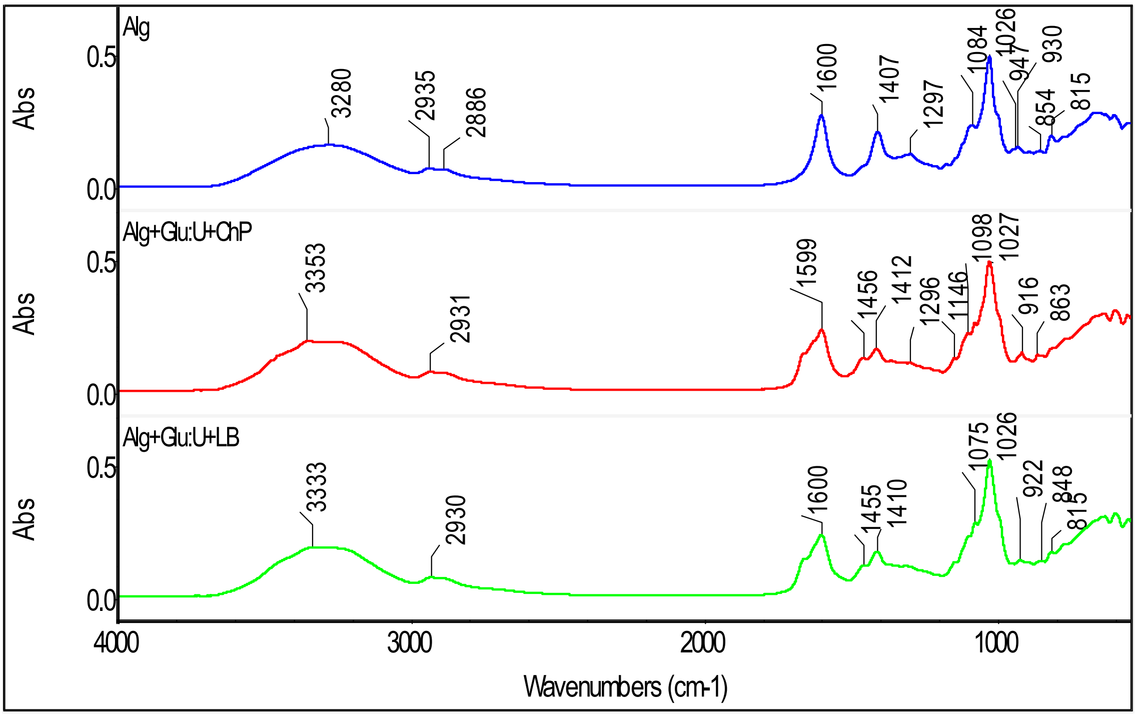

Figure S5. ATR−FTIR spectra of the films: plasticized alginate, Alg+Glu:U+ChP and Alg+Glu:U+LB.
